# Effect of Ag nanoparticle concentration on the electrical and ferroelectric properties of Ag/P(VDF-TrFE) composite films

**DOI:** 10.1038/srep13209

**Published:** 2015-09-04

**Authors:** Haemin Paik, Yoon-Young Choi, Seungbum Hong, Kwangsoo No

**Affiliations:** 1 Department of Materials Science and Engineering, Korea Advanced Institute of Science and Technology, Daejeon, Korea; 2 Materials Science, California Institute of Technology, Pasadena, CA 91125, USA; 3 Materials Science Division, Argonne National Laboratory, Lemont, IL 60439, USA

## Abstract

We investigated the effect of the Ag nanoparticles on the ferroelectric and piezoelectric properties of Ag/poly(vinylidenefluoride-trifluoroethylene) (P(VDF-TrFE)) composite films. We found that the remanent polarization and direct piezoelectric coefficient increased up to 12.14 μC/cm^2^ and 20.23 pC/N when the Ag concentration increased up to 0.005 volume percent (v%) and decreased down to 9.38 μC/cm^2^ and 13.45 pC/N when it increased up to 0.01 v%. Further increase in Ag concentration resulted in precipitation of Ag phase and significant leakage current that hindered any meaningful measurement of the ferroelectric and piezoelectric properties. 46% increase of the remanent polarization value and 27% increase of the direct piezoelectric coefficient were observed in the film with the 0.005 v% of the Ag nanoparticles added without significant changes to the crystalline structure confirmed by both X-ray diffraction (XRD) and Fourier transform infrared (FT-IR) experiments. These enhancements of both the ferroelectric and piezoelectric properties are attributed to the increase in the effective electric field induced by the reduction in the effective volume of P(VDF-TrFE) that results in more aligned dipoles.

Emerging needs for clean and sustainable power sources for wireless sensor networks (WSNs) require research and development of materials, materials integration and design, and fabrication of devices suitable for energy harvesting of ambient sources such as solar, thermal, wind, and vibration energy. Among them, piezoelectric energy harvesters (PEHs) have drawn attention as efficient transduction devices, which convert ambient vibrational energy into usable electrical energy. We expect that PEHs will be implemented into WSNs to provide sustainable power^1^,^2^,^3^,^4^,^5^.

PEHs, in particular, have several advantages over existing power sources that have limited lifetime, and require constant replacement^6^. For example, the wireless sensors for real-time structural health monitoring of bridges, aircrafts, and power plants are essential to prevent catastrophic failure of such infrastructures^7^. The operation of such WSNs requires small and sustainable power source, which can be implemented successfully using PEHs.

Using piezoelectric polymers for PEHs comes with several advantages when applied to WSNs for their flexibility, lightness, and relatively simple manufacturing process. However, the biggest challenge with piezoelectric polymer has been their insufficient power generation for practical applications due to small amount of current produced from PEHs. In order to harness their full potential, it is necessary to develop creative and disruptive materials and structure design at both micro- and nano-scale.

To achieve this, we propose to use conductive silver (Ag) nanoparticles, which are relatively cheap and eco-friendly, as additives to improve the properties of piezoelectric polymer, poly(vinylidenefluoride-trifluoroethylene) (P(VDF-TrFE)), which is one of the widely studied piezoelectric polymers for its high piezoelectric response^8^,^9^.

Most of the studies about the effects of Ag nanoparticles on piezoelectric polymers have focused on the percolation effect of the nanoparticles on the electric properties as well as dialectic properties in terms of energy storage of the polymer^10^,^11^,^12^. However, there is still under controversial debate whether the Ag nanoparticles improves or deteriorates the electric properties^13^,^14^,^15^. Furthermore, there has been no systematic study about the effect of Ag nanoparticles on the piezoelectric properties, which are crucial for applications to PEHs. Therefore, understanding the mechanism behind the change of piezoelectric properties as a function of Ag nanoparticle concentration in P(VDF-TrFE) films remains of particular scientific and technological interest in view of designing a novel piezoelectric polymer based PEHs.

In this study, we present the effect of addition of Ag nanoparticles into P(VDF-TrFE) films and the optimum concentration of Ag nanoparticles to enhance the piezoelectric properties.

## Results and Discussion

**Figure 1:**
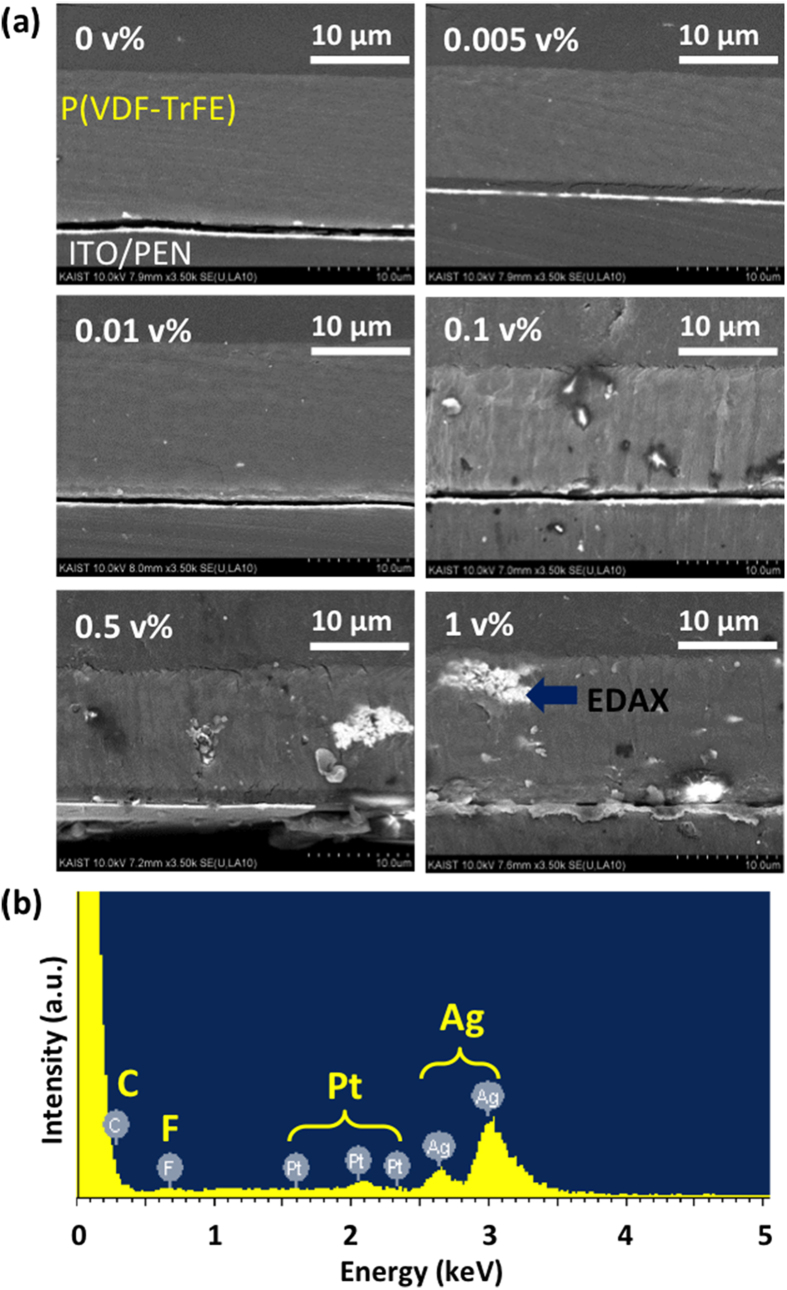
(**a**) SEM images with backscattered electrons detector of films cross-section with Ag concentration form 0 to 1 v%, and (**b**) the EDS spectrum analysis to certify that brighter contrast showed the Ag particles.

Figure 1 shows the cross-section SEM images of the films embedded with Ag nanoparticles from 0 to 1 v%. When more than 0.1 v% of Ag nanoparticles were added to P(VDF-TrFE) films, instead of well dispersing, Ag particles agglomerated together to form larger clusters showing brighter contrast in scanning electron microscopy (SEM) images. These phases are also noticed in optical images of top view of films (see Supplementary Fig. S1 online). Energy-dispersive X-ray spectroscopy (EDS) spectrum analysis indicated that the bright part corresponds to Ag.

**Figure 2:**
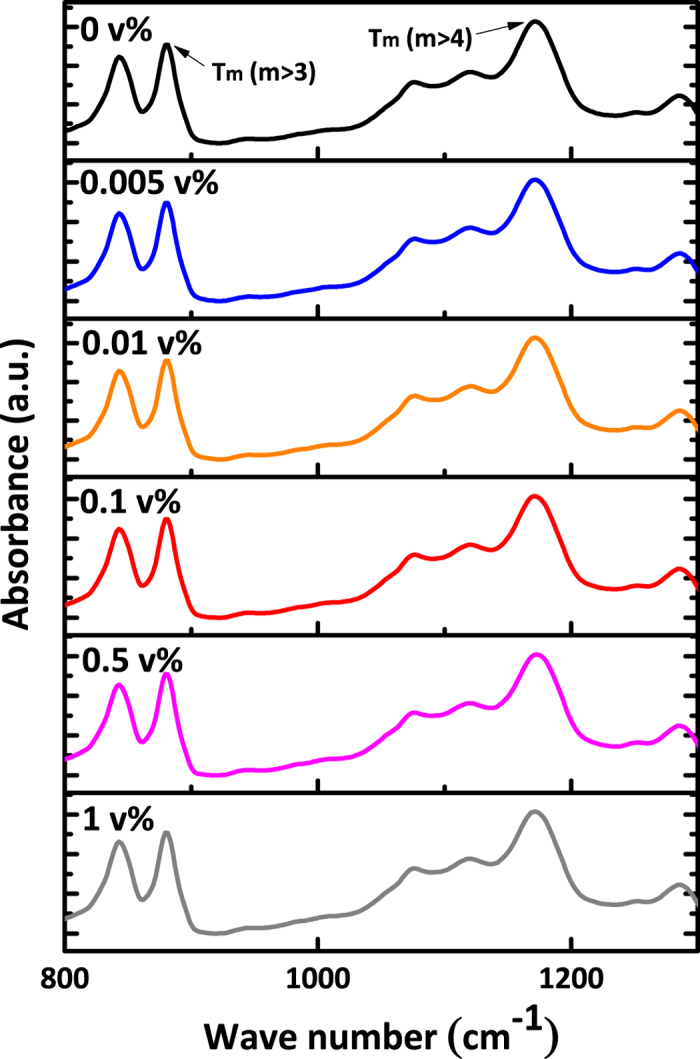
FT-IR spectra of Ag/P(VDF-TrFE) composite films.

Figure 2 displays the FT-IR spectroscopy of P(VDF-TrFE) films, from which we can identify the conformation of the polymer chains in the films. We found almost identical spectra for Ag/P(VDF-TrFE) composite films from 0 to 0.1 v% of Ag nanoparticles concentration. This indicates that addition of Ag nanoparticles in P(VDF-TrFE) films has insignificant effect on the internal structures of the films within the detection limit of FT-IR.

**Figure 3:**
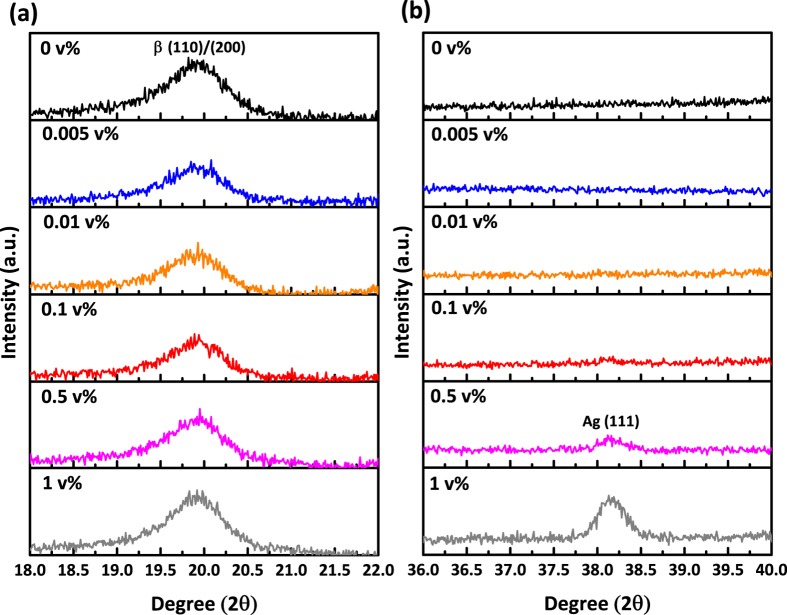
XRD pattern of Ag/P(VDF-TrFE) over different amount of Ag nanoparticles. (**a**) 2θ scan range is from 18° to 22° to examine the characteristic peak from the (110)/(200) planes of P(VDF-TrFE) β phase at 19.7°. (**b**) XRD spectrum is shown in the range between 36° and 40° for Ag (111) peak which is at 38.14°. The intensities for all films are normalized by the peak height from the substrate, which is at 26.9° (Fig. S1).

The XRD patterns shown in Fig. 3 also indicate that the addition of Ag nanoparticles has little impact on the crystallographic orientation of the polymer chain structures in P(VDF-TrFE) films. The intensities of the peak at 19.7° associated with the crystalline (110) and (200) planes of all-trans conformation^16^ are comparable in four different Ag nanoparticles concentration while the (111) planes in Ag at 38.14°^17^ appeared when Ag concentration increased above 0.5 v%. This data agrees with the results shown in Fig. 2 where we found that the addition of Ag nanoparticles does not affect substantially the internal structure of the polymer film.

**Figure 4:**
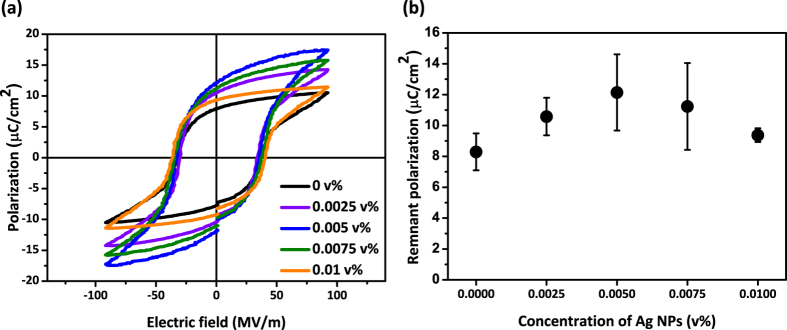
(**a**) P-E loops of films with Ag concentration from 0 to 0.01 v% and (**b**) the remanent polarization values corresponding to each P-E loop. Error bars indicate standard deviation from five different samples.

The polarization vs. electric field (P-E) hysteresis loops for the films from 0 to 0.01 v% of Ag concentration are shown in Fig. 4. Each polarization value was measured as the external electric field was applied from −100 to 100 MV/m. The coercive field (E_c_) values were almost constant in five different concentrations but the remanent polarization values were substantially different. The remanent polarization (P_r_) values indicate the internal dipole moment per unit volume when the applied electric field is zero. The values increased until the concentration of Ag nanoparticles reached 0.005 v%, and decreased with larger nanoparticles concentration above 0.005 v%. When the Ag nanoparticles were added more than 0.01 v% to the films, P-E hysteresis loops were not obtained in the same range of applied electric fields due to the large leakage current through the films.

**Figure 5:**
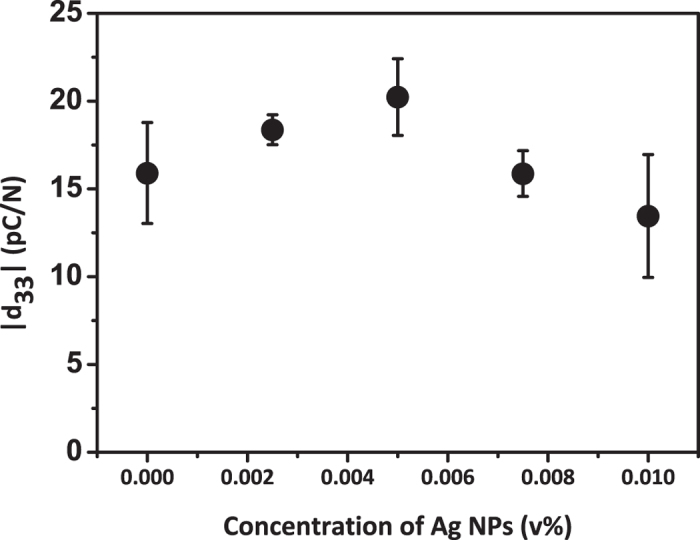
The d_33_ values of the films with Ag concentration from 0 to 0.0 1 v%. Error bars indicate standard deviation from five different samples.

Also the piezoelectric charge coefficient (d_33_) values followed the same trend with the remanent polarization, having the largest value in the 0.005 v% of Ag/P(VDF-TrFE) composite film (Fig. 5). According to Figs 4 and 5, adding 0.005 v% of Ag nanoparticles to the P(VDF-TrFE) film enhances the ferroelectric and piezoelectric properties. This result is also supported by piezoresponse force microscopy (PFM) imaging results, which shows the highest amplitude signal in the 0.005 v% sample (see Supplementary Fig. S4 online)^18^,^19^. Based on our results, we found that 0.005 v% is the optimized condition for the highest piezoresponse of the Ag/P(VDF-TrFE) film.

We attempt to explain the results of P-E hysteresis loops and d_33_ by assuming that there are two competing effects when Ag nanoparticles are added. One is dipole alignment effect and the other is dipole pinning effect. The dipole alignment effect relates to the fact that Ag nanoparticles can act as the center of field concentration that enhances the dipoles to align with the external electric field. In the meantime, the dipole pinning effect refers to the pinning of ferroelectric domain walls at the Ag nanoparticles due to the strong interaction between them, which is commonly observed in ferroelectric ceramic thin films^20^.

**Figure 6:**
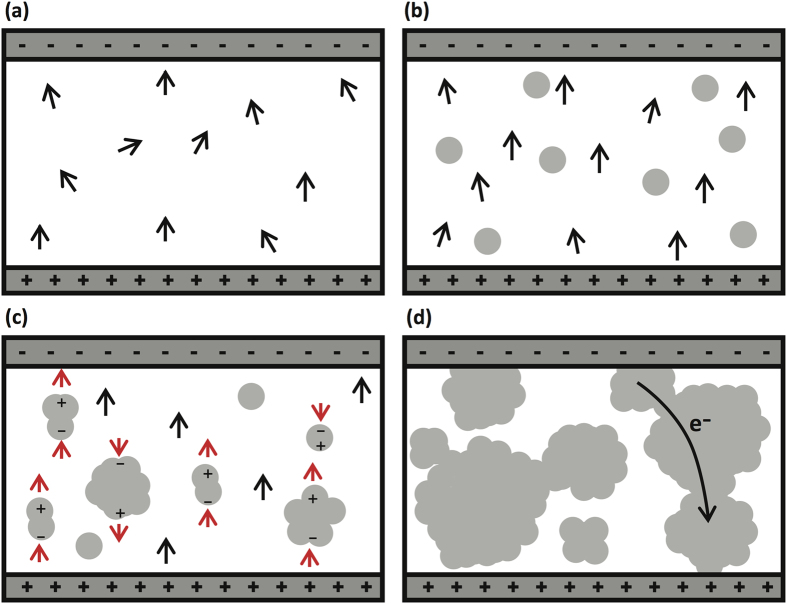
Schematic illustration of dipole alignment effect and dipole pinning effect on the films depending on the amount of the Ag concentration. (**a**) Pure P(VDF-TrFE) film, (**b**) film with Ag concentration less than 0.005 v%, (**c**) film with Ag concentration between 0.005 v% and 0.01 v%, and (**d**) film with Ag concentration above 0.01 v%.

When the electric field is applied to the pure P(VDF-TrFE) film, we think that only a few dipoles will be aligned (Fig. 6(a)) along the electric field due to insufficient electric field to switch all the dipoles in one direction. As we increase the amount of Ag nanoparticles as shown in Fig. 6(b), the imbedded Ag nanoparticles increases the effective electric fields by reducing the effective volume of the polymer in the films. This is the case for Ag particles with less than 0.005 v%. When we increase the concentration above 0.005 v%, however, the dipoles of polymer are pinned by the surface charges or defects at the interface between the matrix and the Ag particles (Fig. 6(c))^21^,^22^. The pinned dipoles cause the decrease in the remanent polarization. However, the coercive voltage remains the same for each film as the field concentration, which tends to decrease the coercive field, and the dipole pinning, which tends to increase the coercive field, compete in the opposite directions.

One more aspect that needs to be considered is the effect of nanoparticle size as the nanoparticles agglomerate as a function of the concentration. It is known that the strength of the field concentration decreases as the size increases. Therefore, the field concentration effect will stop increasing as the Ag nanoparticles start to agglomerate and increase their size as we increase the volume percentage of the Ag nanoparticles. This may explain, in addition to the dipole pinning effect, the decrease of the piezoelectric properties when the concentration increases beyond 0.005 v%. Finally, when we increase the amount of Ag particles further, they will percolate to create conduction path that results in leakage current (Fig. 6(d)).

## Conclusions

We varied the amount of Ag nanoparticles inside the Ag/P(VDF-TrFE) composite films and studied its impact on the piezoelectric properties. We found that adding Ag nanoparticles into the composites had little effects on the internal crystalline structures of polymer chains, which was supported by XRD and FT-IR results. However, the remanent polarization increased from 8.29 to 12.14 μC/cm^2^ when the Ag concentration increased to 0.005 v%, and decreased from 12.14 to 9.38 μC/cm^2^ when the Ag concentration increased to 0.01 v%. Likewise, the film with 0.005 v% of Ag nanoparticles also had the highest piezoelectric charge coefficient values, 20.23 pC/N. We believe that the presence of the nanoparticles up to an optimum amount facilitates the dipole direction to align well under external electric fields, which leads to increase in the polarization of the entire film. On the other hand, the nanoparticles agglomerate to enhance the charge transport, which leads to increase in leakage current and decrease in piezoelectric properties.

## Methods

To make Ag/P(VDF-TrFE) composite solution, dispersed Ag nanoparticles (Ditto Technology Co.) in methyl ethyl ketone (MEK) solvent and 15 weight percent (wt%) of P(VDF-TrFE) (75/25 mol%) were mixed through sonication method for 2 hours. We varied the amount of Ag nanoparticles from 0 to 1 v% in the solution. Films with 15 μm thickness were fabricated by tape casting method^23^. The prepared solution was dropped on to commercially available indium tin oxide (ITO)/polyethylene naphthalate (PEN) films (DuPont, Inc.) and spread by a doctor blade. To increase the crystallinity and align the internal dipoles of the films, we annealed the films in a vacuum oven at 135 °C for an hour. The platinum (Pt) top electrodes were deposited by a dc magnetron sputtering system over shadow mask having 1 mm diameter holes.

The cross-section of the films was observed by SEM with back scattering detector (BSE) mode to distinguish Ag particles clearly and the built-in EDS was used for the element analysis of the samples. Due to low electrical conductivity of P(VDF-TrFE), a few nm thick platinum film was deposited on the cross-section surface for clear SEM image. Characterization of polymer chain structures was conducted by thin film XRD and FT-IR analysis.

The effects of Ag nanoparticles on the ferroelectric properties of P(VDF-TrFE) were studied by measuring polarization hysteresis loops (P-E loops) using the standardized ferroelectric test system, RT66A tester (Radiant technology, Inc.) applying triangular pulses from −100 to 100 MV/m between top and bottom electrodes of the samples. The piezoelectric properties were characterized by measuring piezoelectric coefficient using the d_33_ meter (Piezotest, Inc.) at 110 Hz followed by 15 minute process under 1000 V (66.7 MV/m).

## Additional Information

**How to cite this article**: Paik, H. *et al.* Effect of Ag nanoparticle concentration on the electrical and ferroelectric properties of Ag/P(VDF-TrFE) composite films. *Sci. Rep.* **5**, 13209; doi: 10.1038/srep13209 (2015).

## Supplementary Material

Supplementary Information
