# Supplementary material for: Effect of Ag nanoparticle concentration on the electrical and ferroelectric properties of Ag/P(VDF-TrFE) composite films
Source: Sci Rep. 2015 Sep 4;5:13209. doi: 10.1038/srep13209 (PMC4559747; doi:10.1038/srep13209)
Supplement: Supplementary Information [file srep13209-s1.pdf]

Supplementary Information for

Effect of Ag nanoparticle concentration on the electrical and ferroelectric properties of Ag/P(VDF-TrFE) composite films

Haemin Paik[1,2], Yoon-Young Choi[3], Seungbum Hong[1,3*], Kwangsoo No[1*]

[1]*Department of Materials Science and Engineering, Korea Advanced Institute of Science and Technology, Daejeon, Korea*

[2]*Materials Science, California Institute of Technology, Pasadena, CA 91125*

[3]*Materials Science Division, Argonne National Laboratory, Lemont, IL 60439*

Optical microscopic images of Ag/P(VDF-TrFE) film

Due to transparency of P(VDF-TrFE) and ITO/PEN substrate, distribution of Ag nanoparticles over the polymer films was observable through the optical microscopy. Figure S1 shows the optical microscopic images of films taken from the top of them having different loading amount of Ag nanoparticles from 0.005 v% to 0.5 v%. It is noticed that Ag nanoparticles tend to agglomerate to reduce the surface interaction between Ag and P(VDF-TrFE) rather be separated each other in the polymer matrix. These configuration affects the electric conductivity and dielectric properties of composite films.

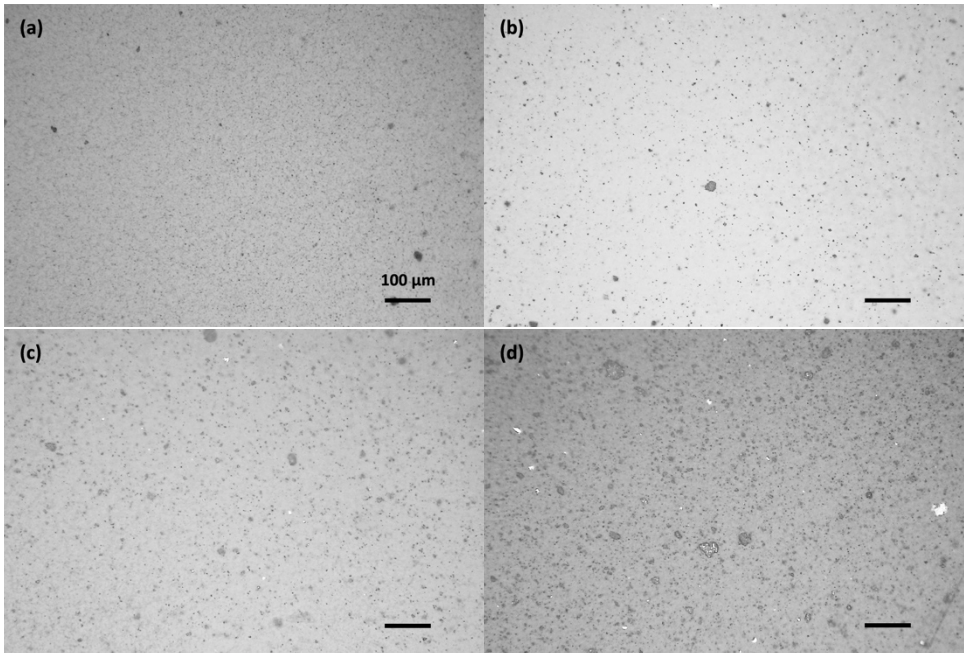

Fig. S1. Optical microscopic images of top view of Ag/P(VDF-TrFE) composite films. Each amount of loading Ag nanoparticles is (a) 0.005 v%, (b) 0.05 v%, (c) 0.1 v%, and (d) 0.5 v%.

X-ray diffraction (XRD) patterns in full range

XRD patterns of each sample were acquired by 2 theta scanning from 18° to 40° and all the XRD spectra heights were normalized by the substrate peak intensity. The diffraction signals from the ITO/PEN substrate is located at 26.9° with intensity exceeding the peaks of beta phase of polymer and Ag nanoparticles, so that those two peaks are hardly noticed. Figure S2 shows the XRD pattern of deposited ITO on the PEN film and the one of the 1 v% Ag nanoparticles imbedded P(VDF-TrFE) film to visualize the relative intensities of

the substrate and two peaks we are interested in.

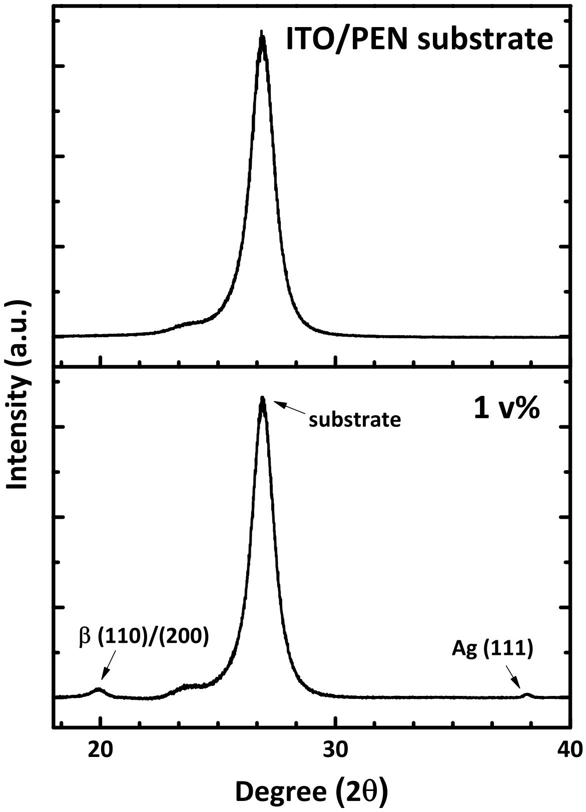

Fig. S2. XRD patterns of the ITO/PEN substrate and the P(VDF-TrFE) film with 1 v% Ag nanoparticles on the ITO/PEN substrate.

Experiment condition of PFM measurements μm

Surface topography, piezoresponse amplitude and phase images were measured by dual AC resonance tracking PFM (DART-PFM)[1] mode (MFP-3D AFM, Asylum Research). A high frequency modulating voltage (200 - 400 kHz, 2.0 V) was applied to the film surface through the Pt/Ir coated Si cantilever (radius of 30 nm, spring constant $K \approx 2.8$ N/m, PPP-

EFM, Nanosensors) during 256 lines scans with scan rate of 1 Hz.

Topography, piezoresponse amplitude and phase images by PFM

To understand the effect of Ag nanoparticles in the P(VDF-TrFE) film in micro-scale, PFM study was conducted to obtain piezoresponse amplitude and phase images. As shown in Figure S3, there are noticeable distinctions in three different films in piezoresponse amplitude and phase images. The average magnitude of amplitude is indicated graphically in Figure S4. The highest piezoresponse amplitude appears in the 0.005 v% Ag/P(VDF-TrFE) film, which represents it has the most amount of electric dipoles responding to external electric field. This study supports our interpretation in micro-scale that 0.005 v% concentration of Ag nanoparticles maximizes the piezoresponse of P(VDF-TrFE) film. However, further study is required for understanding piezoresponse phase images that we will conduct in the future.

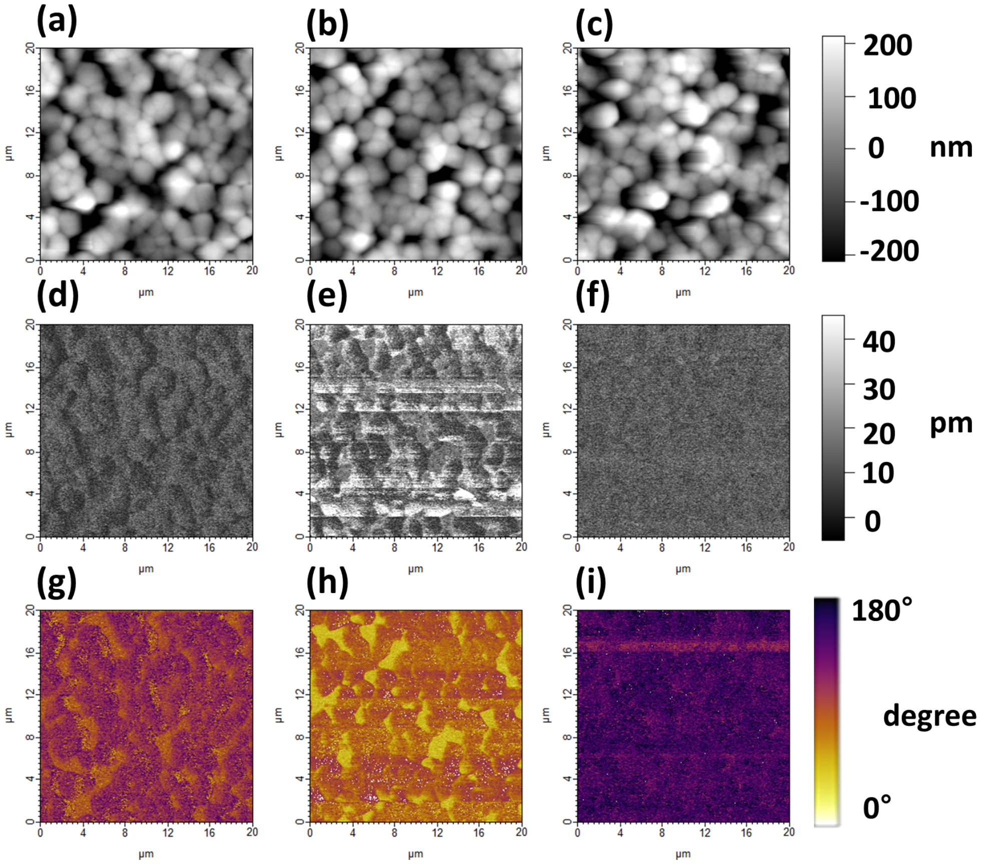

Fig. S3. (a), (b) and (c) are topography images, (d), (e) and (f) are piezoresponse amplitude images, and (g), (h) and (i) are piezoresponse phase images. (a), (d), and (g) are images from pure P(VDF-TrFE) film, (b), (e) and (h) are from 0.005 v% Ag of Ag/P(VDF-TrFE) composite film, and (c), (f) and (i) are from 0.01 v% of Ag/P(VDF-TrFE) composite film. The scale of all images is 20μm by 20μm.

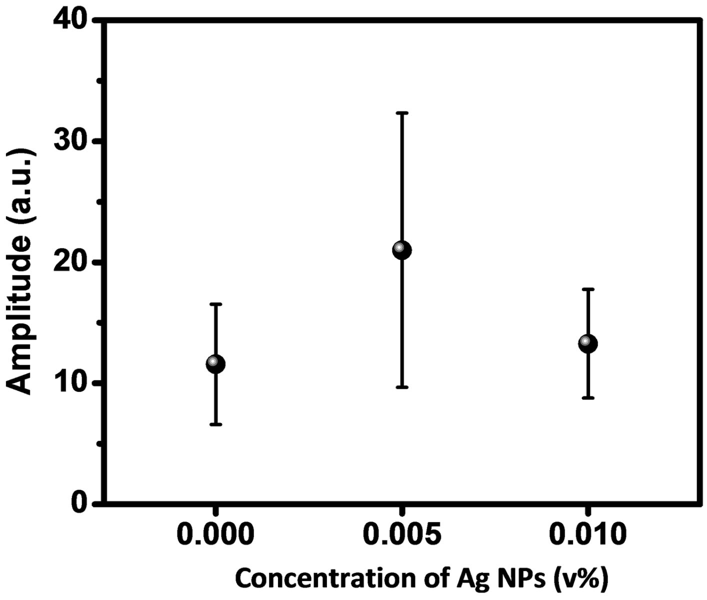

Fig. S4. Average of piezoresponse amplitude from scanned area of 0, 0.005 and 0.01 v% Ag/P(VDF-TrFE) composite films

Size of the Ag nanoparticles

The average size of the purchased Ag nanoparticles was stated as 100 nm. The actual size was determined as 130 ± 110 nm by measuring the 20 nanoparticles based on the SEM images of Ag nanoparticles, which were dispersed in Acetone and casted on silicon substrate, and averaging them (Figure S5). The average crystalline size could be also determined by the Scherrer equation, $t \sim 0.9\lambda/\beta\cos\theta$, where t is the mean size of the crystalline grains, β is the full width at the half maximum (FWHM) intensity at $2\theta = 38.14°$, and λ is X-ray wavelength, which is 0.1541nm for Cu Kα. Using the FWHM of the (111) peak extracted from the Lorentzian fitting, the crystalline size was calculated as 25 nm, which matched to measured size. This result implies that Ag nanoparticles are polycrystalline with around 25 nm size of grains.

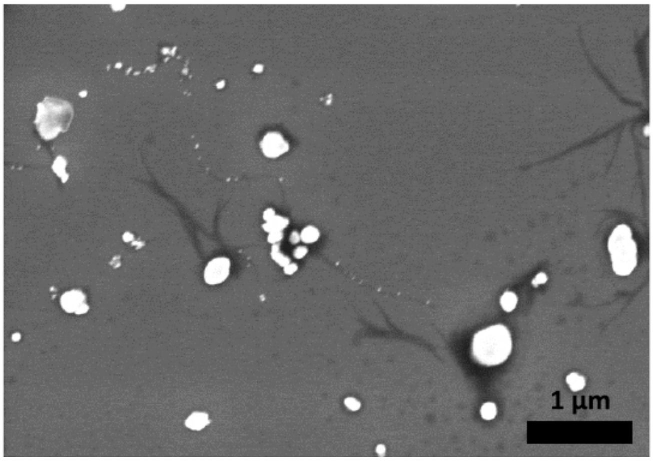

Fig. S5. SEM image of Ag nanoparticles casted on silicon substrate.

Electric properties of Ag/P(VDF-TrFE) films

We studied the general electric properties of Ag/P(VDF-TrFE) composite films to understand their piezoelectric and ferroelectric properties. Relationship between the current density and applied voltage in four different films with volume fraction of Ag nanoparticles from 0 to 0.1 v% was investigated using four point probes connected to the HP 4156A Precision Semiconductor Parameter Analyzer (Figure S6). DC voltage was swept from 0 V to 20 V with 0.5 V step voltage. While the resistance, which is the slope of the curve, remained almost the same from 0 v% to 0.01 v% of Ag nanoparticles content, the film with the 0.1 v% of Ag showed much smaller resistance than the others with an Ohmic behaviour in response to the external applied voltage. As such, we were not able to study the ferroelectric and piezoelectric properties of the films with more than 0.1 v% of Ag nanoparticles.

Effective capacitance, dielectric constant and tan δ for each film were also measured using d_{33} meter and the test frequency was 1 kHz. As shown in Figure S7 (a), it is a well-known phenomenon that embedding nanocomposites such as Ag to the dielectric polymer increases dielectric constant values in accordance with the universality percolation theory. It does not follow the power law behaviour here due to agglomerated Ag nanoparticles in high concentration as shown in Fig. S1. Due to the non-uniform spatial distribution of percolated Ag nanoparticles, the error bar increases with the volume fraction of Ag nanoparticles in the composites. The dielectric loss, which is represented by tan δ in Figure S7 (b), increases as the amount of Ag nanoparticles increases due to the higher conductivity. It is noted that small value of tan δ less than 0.05 allows for reliable measurement of dielectric properties, which was the case for the composites with Ag nanoparticles concentration of up to 0.1 v%.

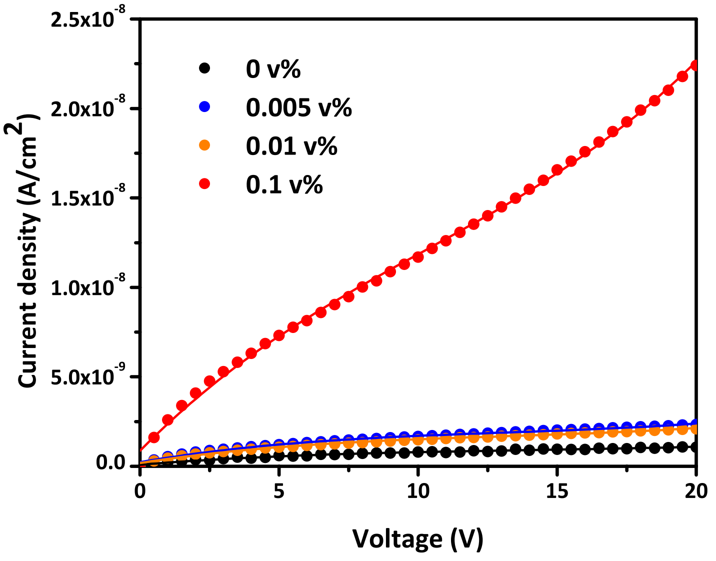

Fig. S6. I-V curves of Ag/P(VDF-TrFE) composite films with the volume fraction of Ag nanoparticles from 0 to 0.1.

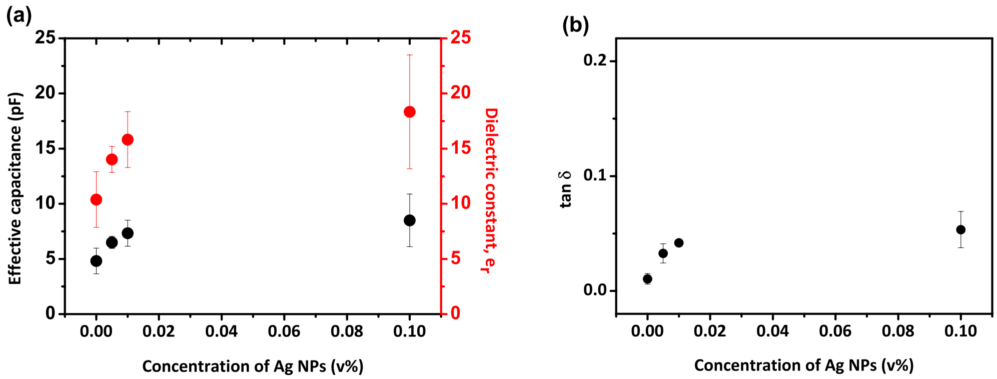

Fig. S7. Dielectric properties change over amount of Ag nanoparticles filling. (a) Effective capacitance and dielectric constant values and (b) tangent δ values in function of volume fraction of Ag nanoparticles.

1. Rodriguez, B. J., Callahan, C., Kalinin, S. V. & Proksch, R. Dual-frequency resonance-tracking atomic force microscopy. *Nanotechnology* **18,** 475504 (2007).
